# Resting-State Default Mode Network Related Functional Connectivity Is Associated With Sustained Attention Deficits in Schizophrenia and Obsessive-Compulsive Disorder

**DOI:** 10.3389/fnbeh.2018.00319

**Published:** 2018-12-19

**Authors:** Jie Fan, Jun Gan, Wanting Liu, Mingtian Zhong, Haiyan Liao, Hongchun Zhang, Jinyao Yi, Raymond C. K. Chan, Changlian Tan, Xiongzhao Zhu

**Affiliations:** ^1^Medical Psychological Center, The Second Xiangya Hospital, Central South University, Changsha, China; ^2^Medical Psychological Institute of Central South University, Changsha, China; ^3^Center for Studies of Psychological Application, School of Psychology, South China Normal University, Guangzhou, China; ^4^Department of Radiology, The Second Xiangya Hospital, Central South University, Changsha, China; ^5^Neuropsychology and Applied Cognitive Neuroscience Laboratory, CAS Key Laboratory of Mental Health, Institute of Psychology, Chinese Academy of Sciences, Beijing, China; ^6^Department of Psychology, University of Chinese Academy of Sciences, Beijing, China

**Keywords:** sustained attention, sustained attention to response task, resting-state functional connectivity, default mode network, salience network, frontal-parietal network

## Abstract

**Background:** Previous studies have indicated the resting-state default mode network (DMN) related connectivity serving as predictor of sustained attention performance in healthy people. Interestingly, sustained attention deficits as well as DMN-involved functional connectivity (FC) alterations are common in both patients with schizophrenia (SCZ) and with obsessive-compulsive disorder (OCD). Thus, the present study was designed to investigate whether the DMN related resting-state connectivity alterations in these two psychiatric disorders were neural correlates of their sustained attention impairments.

**Methods:** The study included 17 SCZ patients, 35 OCD patients and 36 healthy controls (HCs). Sustained attention to response task was adopted to assess the sustained attention. Resting-state scan was administrated and seed-based whole-brain FC analyses were performed with seeds located in classical DMN regions including bilateral medial prefrontal cortex (mPFC) and posterior cingulate cortex (PCC).

**Results:** Both SCZ and OCD patients had poorer sustained attention than HCs. Sustained attention deficits in OCD was negatively correlated with their impaired FC of right mPFC-left superior frontal gyrus (SFG) within DMN, and that in SCZ was significantly correlated with their altered FC of left mPFC-bilateral anterior cingulate cortex (ACC) which indicated interaction between DMN and salience network. In addition, the FC between left mPFC and right parietal lobe indicating the interaction between DMN and frontal-parietal network was correlated with sustained attention in both SCZ and OCD.

**Conclusion:** These findings suggest the importance of DMN-involved connectivity, both within and between networks in underlying sustained attention deficits in OCD and SCZ. Results further support the potential of resting-state FC in complementing information for cognitive deficits in psychiatric disorders.

## Introduction

Sustaining a moderate level of attention is essential for the performance of many everyday activities. Deficits in sustained attention, however, are very common in patients with schizophrenia (SCZ) and patients with obsessive-compulsive disorders (OCD; Lee et al., 2009; Hong et al., 2011). Both SCZ and OCD are recognized as neurodevelopmental disorders (Tibbo and Warneke, 1999). OCD occurs in a proportion of 14% of the SCZ patients, and the transitions from OCD to SCZ can be clinically observed (Swets et al., 2014; Scotti-Muzzi and Saide, 2018). Several previous studies have revealed the overlapping structural and functional brain dysfunction in SCZ and OCD, indicating the somewhat shared pathomechanisms across these two psychiatric conditions (Tibbo and Warneke, 1999; Gross-Isseroff et al., 2003). Based on these links between SCZ and OCD, it may be of great interest to explore some common cognitive deficits in these two disorders, in terms of which, for example, the sustained attention impairments.

Continuous performance tasks (CPTs) as well as its variants, such as the sustained attention to response task (SART) are the main assessments of the sustained attention function. During these tasks, participants are required to respond to most trials, while stop on minority of target trials (Esterman et al., 2013). The trial-to-trial reaction time (RT) variation, also known as the intra-individual variability (IIV), indicating the attention fluctuation level among participants, serves as the most sensitive variable for the measurement of the sustained attention. Using these paradigms, researchers have revealed substantial evidence showing sustained attention deficits in SCZ and OCD (Hong et al., 2011; Abramovitch et al., 2013). However, despite these preliminarily established behavioral impairments, the neurobiological source of these deficits remains not well understood.

Task functional magnetic resonance imaging (fMRI) is usually the most popular way to investigate the neural mechanism of the cognitive dysfunctions. However, studies have revealed that some of the variability in the task-based brain changes might actually due to the spontaneous fluctuations that made up the resting-state data (Fox and Raichle, 2007). If the brain areas have impaired connectivity at baseline, performance during behavioral tasks which engage these regions may be compromised. Due to these notions, recently, utilizing resting-state fMRI to study the disease-related variability that complements changes observed due to cognitive tasks has gained increasing attention. Notably, previous studies using the resting-state methods have indicated that the function connectivity involved default mode network (DMN) in the resting-state can serve as the predictors of sustained attention (Bonnelle et al., 2011; Christakou et al., 2013).

Default mode network refers to a collection of brain regions showing consistently greater activity during the resting state. Core hubs of DMN are the medial prefrontal cortex (mPFC) and the posterior cingulate cortex (PCC; Raichle and Snyder, 2007) and it is usually thought to be related to the self-referential processing. Also, it should be noted that as the typical task-negative network, DMN has strong anti-relationship with the task-positive networks, including the central executive network (CEN) and the salience network (SN; Raichle and Snyder, 2007; Menon, 2011). The interactions between DMN and these task-positive networks are also important for the brain function (Sridharan et al., 2008). Disruptions of the DMN as well as the connectivity between DMN and other networks are clinically important, and abnormalities have been observed in varying psychiatric disorders including SCZ (Hu et al., 2017) and OCD (Fitzgerald et al., 2010).

Hypothesis that DMN related activities at the resting-state may provide prospection for the performance during sustained attention tasks are suggested by the following evidence. First, it has been suggested by results from the resting-state studies among healthy people (Kelly et al., 2008). Second, previous studies have showed that the failure to suppress activity in DMN both at the beginning of the tasks and during the tasks tracked the momentary attention lapses (Bonnelle et al., 2011; Christakou et al., 2013). Since it has been revealed that findings of connectivity at the resting-state carried somewhat similar predictive function for cognitive performance as connectivity during the tasks (Hampson et al., 2006; Kelly et al., 2008), the hypothesis that DMN activity at rest could also provide information for sustained attention was plausible. Third, theoretically, the DMN activities, either at the rest or during the tasks, might be related to the occurrence of the stimulus-independent thoughts and the mind-wandering, which were known as the decisive factors for attention lapses during sustained attention (Mason et al., 2007; Hasenkamp et al., 2012). Hence, to conclude, these results all suggest the potential role of the resting-state DMN-involved FC in providing the information for sustained attention performance.

Given these aforementioned inspirations, as well as the findings that the DMN-involved FC abnormalities and the sustained attention deficits were both observed in SCZ and OCD, an interesting question was raised on whether the DMN related abnormalities were makers of the sustained attention deficits across different mental disorders. The present study was designed to investigate this issue. To do so, we (1) examined the associations between the altered DMN related FC and the sustained attention deficits in SCZ and OCD separately; (2) examined whether there was common FC correlated with sustained attention in both SCZ and OCD. Sustained attention was assessed by SART, and the DMN related connectivity was explored using the resting state seed-based FC method. Our hypothesis was that the resting-state DMN involved connectivity could serve as neural correlates of sustained attention deficits across different mental disorders.

## Materials and Methods

### Participants

Nineteen SCZ patients and 36 OCD patients participated in the present study. They were all outpatients recruited from the psychological clinic at The Second Xiangya Hospital. Data from three patients (2 SCZ and 1 OCD) were excluded from final analysis due to excessive head motion during the imaging scan (see Materials and Methods).

All included SCZ patients satisfied the DSM-V criteria for SCZ (American Psychiatric Association [APA], 2013). The severity of schizophrenic symptoms indicated by the scores of the Positive and Negative Syndrome Scale (PANSS, Kay et al., 1987) was 58.71 ± 12.10 and the mean illness duration was 19.94 ± 22.67 months. Of the included 17 patients, 5 were drug-naïve, 1 did not take psychotropic medicines for 6 months, and the remaining 11 were under antipsychotic medication at the time of testing (paliperidone extended-release: *n* = 1, 6 mg/day; quetiapine: *n* = 2, 25–75 mg/day; risperidone: *n* = 3, 2–5 mg/day; olanzapine: *n* = 5, 5–10 mg/day).

The included 35 OCD patients all met the DSM-V criteria for OCD (American Psychiatric Association [APA], 2013). Illness severity suggested by the scores of Yale-Brown Obsessive-Compulsive Scale (Y-BOCS; Goodman et al., 1989) was 30.37 ± 6.14 and the mean illness duration was 63.71 ± 66.94 months. Of the 35 OCD patients, 18 were treatment naïve, 2 did not take psychotropic medications for a minimum of 3 months, and the remaining 15 were under medication at the day of scan (sertraline: *n* = 13, 50–200 mg/day; paroxetine: *n* = 1, 4 mg/day; citalopram: *n* = 1, 20 mg/day). Exclusion criteria for both SCZ and OCD patients were: (1) axis I psychiatric disorder comorbidity; and (2) history of major medical or neurological problems. The diagnosis and comorbidities for each patient were established by the consensus between two experienced psychiatrists according to the Structured Clinical Interview for the DSM-V Axis I (SCID-I).

Thirty-six students or staff members at the Central South University were recruited as healthy controls (HCs). The exclusion criteria were: (1) a history of any psychiatric illnesses; and (2) any major medical or neurological problems.

All participants were right-handed, 16–35 years of age, and had at least 9 years education. They also finished the Edinburgh Handedness Inventory (EHI; Oldfield, 1971), Beck Depression inventory (BDI; Beck et al., 1961) and the State Trait Anxiety Inventory (STAI; Spielberger et al., 1983) to determine their handedness, depression and anxiety levels. The Ethics Committee of The Second Xiangya Hospital of Central South University approved this study and all the subjects signed written consent forms before their enrollments.

### Sustained Attention Assessments: SART

Before scanning, all subjects performed the SART test which was programmed by E-prime software. Participants were seated 60 cm from the computer screen in a quite environment to perform it.

For the SART, a total of 225 trials were presented. On each trial, a single digit (1–9) were presented for 250 ms and then followed by a 900 ms mask (a ring with a diagonal cross in the middle, diameter = 2.5 cm). Participants were instructed to respond to the appearance of each digit by pressing the left mouse button (“Go” target), except when the digit was 3 (“No-go” target, 25 of the 225 trials, during which, they needed to withdraw from responding). Accuracy and speed were required to give the equal importance in this task. Each digit has five different font sizes variants (18, 26, 36, 66, and 80 point) to enhance the processing demand for numerical value and to avoid the participants’ use of cognitive strategy of simply searching templates for some peripheral features (Molenberghs et al., 2009). All five kinds of variants for the nine digits were repeated five times during the whole experiment with the orders of digits as well as the font sizes were randomized for each participant. Digits and masks were presented in black bold fonts on the white computer screen. Before the formal SART task, each participant had to perform 8 practice trials, of which two were no-go digit “3.” Practice procedure could be restarted in the case that the participants didn’t understand the task after the first practice.

At the end of the task, the omission error [i.e., failures to respond to the go digit (not “3”)], commission error [i.e., failures to withhold to no-go digit (“3”)], mean reaction time (RT) for correct go trials and the trial-to-trial RT fluctuation (reflected by IIV, defined as RT standard deviation/mean RT) were calculated for each participant.

### Imaging Procedures

#### Image Acquisition

Resting-state fMRI images as well as the three-dimensional T1-weighted, magnetization-prepared rapid gradient echo (MPRAGE) sagittal images which collected for the normalization purpose were acquired on a Siemens Skyra 3-T magnetic resonance scanner at The Second Xiangya Hospital of Central South University. For the acquisition of resting state data, participants were instructed to remain still with eyes closed, and to think of nothing in particular but to avoid falling asleep. The echoplanar imaging sequence was used and the parameters were: 2500-ms repetition time (TR), 25-ms echo time (TE), 39 axial slices, 3.5-mm slice thickness, no gap, 3.8 × 3.8 × 3.5-mm voxel size, 200 volumes, 90° flip angle, 240-mm field of view, and 64 × 64 data matrix. Parameters for the three-dimensional T1-weighted, MPRAGE sagittal images were: 1900-ms TR, 2.01-ms TE, 176 slices, 1.00-mm slice thickness, 1.0 × 1.0 × 1.0-mm voxel size, 9° flip angle, 900-ms inversion time, 256-mm field of view, and 256 × 256 matrix.

#### Image Preprocessing

Data Processing Assistant for Resting-State fMRI software (DPARSF V2.3; Yan and Zang, 2010) was used to preprocess the imaging data. After discarding of first 10 volumes, slice timing and realignment of head motion, data from three subjects (2 SCZ and 1 OCD) were excluded from the further analysis because their translation or rotation exceeded ±1.0 mm or ±1.0°. The remaining images were spatially normalized to the standard Montreal Neurological Institute (MNI) atlas space with resampling to a voxel size of 3 × 3 × 3 mm. Smoothing (Gaussian kernel of 8 × 8 × 8 mm full-width at half maximum) and linear detrending were then conducted. We regressed out nuisance signals involving six head motion parameters, white matter signal, global signal and cerebrospinal fluid signal, and finally, temporal band-pass filtering (0.01–0.08 Hz) was performed.

#### Motion Management and Seed-Based Connectivity

To assess and manage the effects of head motion, we calculated the frame-wise displacement (FD) from the translation and rotation parameters for each subject, and censored (scrubbing) any frames with FD > 0.5 mm (Power et al., 2012). After the scrubbing, the seed-based whole brain FC maps for each participant were calculated. Based on previous studies (Fox et al., 2005), three classical DMN seeds, with each defined as sphere (6-mm radius) centered on the following coordinates (MNI) [PCC (0, –33, 40), left mPFC (–2, 43, –11), and right mPFC (2, 61, 13)] were selected. These seed regions were adopted because of their key roles in the DMN and their reliable identification of the DMN in previous studies (Fox et al., 2005; Gaffrey et al., 2012; Posner et al., 2013).

### Statistical Analysis

To examine whether the altered DMN related connectivity was neural correlate of sustained attention deficits in OCD and SCZ sample independently, we firstly adopted the design of SCZ vs. HC comparison, and the OCD vs. HC comparison separately. Differences in demographic, clinical variables, motion parameters as well as SART performance between two groups were examined using two sample *t*-tests, Mann–Whitney *u*-tests or the Chi-square tests according to the variable type and normality. For the FC maps elicited by each DMN seed, the two-sample *t*-tests were performed on the Statistical Parametric Mapping (SPM V12) to examine the group difference between SCZ and HC and between OCD and HC. Age, gender, and education level were controlled as covariates for all image comparisons. Significance threshold was set at voxel level *p* < 0.005 uncorrected and cluster level *p* < 0.05 false discovery rate (FDR) corrected. When group differences for DMN-involved FCs and SART performance were established, correlation analyses were performed to examine the relationships between the altered DMN related FCs and the impaired sustained attention (IIV) in SCZ and OCD, respectively, with the demographic, clinical and head motion variables which showed significant correlations with the altered FCs and/or IIVs controlled as covariates.

To examine whether there was transdiagnostic FC correlated with sustained attention in both psychiatric conditions, additional regression models among the whole patient sample were run on the SPM. For the regression analysis of each seed, IIV was entered as the dependent variable, and the FC maps were the predictors. Disease type (categorial: SCZ, OCD) was controlled as the covariate. Significance threshold was also set at *p* < 0.005 voxel level uncorrected and *p* < 0.05 cluster level FDR corrected.

## Results

### Comparisons of Demographical, Clinical and Head Motion Variables Between Patient Groups and HCs

Normality tests for all variables by group can be found in Supplementary Table S1. Only omission errors in OCD and HC violated the normal distribution (*ps* < 0.05). Demographic, clinical and head motion information for the three groups are presented in Table 1. Both SCZ and OCD were matched in age and gender with HCs (*ps* > 0.05). OCD patients had similar (*p* > 0.05), while the SCZ patients had lower educational level relative to HCs (*p* < 0.05). Both patient groups demonstrated higher scores of BDI, STAI-S, and STAI-T than HCs (*ps* < 0.05). After scrubbing, all subjects had at least 74% of the frames remained to be calculated which satisfied the analyzable requirements (60%) outlined by Power et al. (2012). The head motion parameters, i.e., the mean FD and the percentage of censored frames, were not differed between both patient groups and HCs (*ps* > 0.05).

**Table 1:** Demographic, clinical variables and behavioral performance during SART for SCZ and OCD samples, respectively.

Samples	SCZ_(*N* = 17)_ vs. HC_(*N* = 36)_					OCD_(*N* = 35)_ vs. HC_(*N* = 36)_				
	SCZ mean (*SD*)	HC mean (*SD*)	*t*/χ^2^/*u*^1^	*p*	*Cohen’s d*	OCD mean (*SD*)	HC mean (*SD*)	*t*/χ^2^/*u*^1^	*p*	*Cohen’s d*
**Demographic and clinical variables**										
Age (years)	22.41 (3.74)	22.86 (2.67)	−0.44	0.661	–	23.86 (5.48)	22.86 (2.67)	0.97	0.338	–
Sex (female, %)	8 (47.1)	23 (63.9)	1.35	0.246	–	16 (45.7)	23 (63.9)	2.37	0.124	–
Education (years)	13.76 (2.54)	15.89 (1.91)	−3.07	<0.01	–	14.96 (2.32)	15.89 (1.91)	−1.85	0.069	–
Duration (month)	19.94 (22.67)	–	–	–	–	63.71 (66.94)	–	–	–	–
Under treatment (medication, %)^∗^	11 (64.7)	–	–	–	–	15 (42.9)	–	–	–	–
PANSS_Total	58.71 (12.10)	–	–	–	–	–	–	–	–	–
PANSS_P	17.47 (4.14)	–	–	–	–	–	–	–	–	–
PANSS_N	10.29 (3.55)	–	–	–	–	–	–	–	–	–
PANSS_G	30.94 (7.16)	–	–	–	–	–	–	–	–	–
YBOCS_Total	–	–	–	–	–	30.37 (6.14)	–	–	–	–
YBOCS_O	–	–	–	–	–	16.40 (2.77)	–	–	–	–
YBOCS_C	–	–	–	–	–	13.97 (4.93)	–	–	–	–
BDI	18.12 (9.69)	5.69 (5.01)	4.98	<0.001	–	20.06 (9.83)	5.69 (5.01)	7.72	<0.001	–
STAI-T	53.00 (10.52)	40.17 (9.08)	4.57	<0.001	–	57.91 (8.72)	40.17 (9.08)	8.40	<0.001	–
STAI-S	50.12 (13.72)	41.33 (8.09)	2.45	<0.05	–	52.17 (11.52)	41.33 (8.09)	4.60	<0.001	–
FD	0.10 (0.06)	0.08 (0.05)	1.32	0.190		0.10 (0.05)	0.08 (0.05)	1.51	0.134	–
Frames censored (%)	0.02 (0.02)	0.01 (0.03)	1.35	0.183		0.01 (0.02)	0.01 (0.03)	0.67	0.507	–
**Behavioral performance during SART**										
Omission error	0.04 (0.04)	0.01 (0.01)	102.50	<0.001	1.029	0.03 (0.04)	0.01 (0.01)	418.50	<0.01	0.686
Commission error	0.48 (0.23)	0.30 (0.15)	3.02	<0.01	0.927	0.40 (0.25)	0.30 (0.15)	2.18	<0.05	0.485
Mean RT (ms)	401.62 (77.05)	402.94 (93.34)	−0.05	0.960	–	393.48 (104.64)	402.94 (93.34)	−0.40	0.689	–
IIV	0.34 (0.08)	0.23 (0.06)	5.47	<0.001	1.556	0.29 (0.12)	0.23 (0.06)	2.68	<0.01	0.632

*^∗^The notion that patients were not under medication treatment means that the patients were medication-naïve, or they did not take any antipsychotic meds at least for 3 months prior to the day when they were enrolled. SCZ, schizophrenia; OCD, obsessive-compulsive disorder; HC, healthy controls; SART, sustained attention to response task; SD: standard deviation; YBOCS, Yale-Brown Obsessive-Compulsive Scale; PANSS, Positive and Negative Syndrome Scale; BDI, Beck Depression Inventory; STAI, State Trait Anxiety Inventory; RT, reaction time; IIV, intra-individual variation; FD, frame-wise displacement. ^1^Comparison results of age, education, commission error, mean RT, IIV, FD and frames censored were indicated by t; results of sex were indicated by χ^2^; comparison results of omission error were indicated by u.*

**FIGURE 1:**
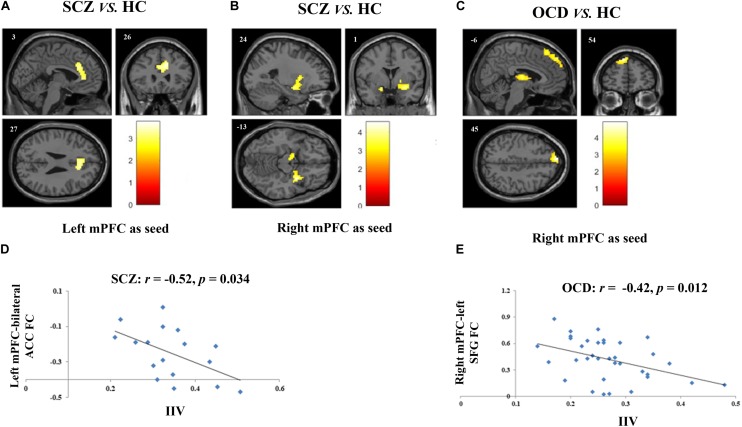
Altered FCs and their correlations with sustained attention deficits in patients with SCZ and with obsessive-compulsive disorder (OCD). **(A)** Two-sample *t*-test revealed that SCZ patients had reduced FC between left mPFC and bilateral ACC as compared with HCs. **(B)** SCZ patients had reduced FC between right mPFC and bilateral amygdala as compared with HCs. **(C)** Results of two-sample *t*-test showed that when the right mPFC as seed, OCD patients had reduced FC in left SFG and in bilateral thalamus as compared with HCs. **(D)** Scatter plots demonstrated that the reduced functional connectivity between left mPFC and bilateral ACC in SCZ group was negatively correlated with their enhanced intra-individual RT variations during SART. **(E)** Scatter plots showed that reduced functional connectivity between right mPFC and left SFG in OCD group was negatively correlated with their sustained attention deficits. For all image analyses, age, gender and education were controlled as covariates. Significance threshold was set at *p* < 0.05, FDR cluster level corrected, starting from voxel level *p* < 0.005 uncorrected. FC, functional connectivity; SCZ, schizophrenia; OCD, obsessive-compulsive disorder; HC: healthy control; SART, sustained attention to response task; IIV, intra-individual variation; mPFC, medical prefrontal cortex; ACC, anterior cingulum cortex; SFG, superior frontal gyrus.

### Comparisons of Behavioral Performance During SART Between Patient Groups and HCs

Detailed performances during SART are summarized in Table 1. Both SCZ and OCD committed more omission and commission errors than HCs (*ps* < 0.05). The two patient groups did not differ with HCs in terms of the mean RT (*ps* > 0.05), while they both showed greater IIV than HCs (*ps* < 0.01). Except for correlation between commission errors and illness duration in SCZ group (*r* = –0.62, *p* < 0.01), no other correlations between altered measurements of sustained attention and demographical, clinical and head motion variables were significant (*ps* > 0.05, Supplementary Table S2).

### Comparisons of Seed-Base Whole Brain FCs Between Patient Groups and HCs

Two-sample *t*-tests showed that SCZ had reduced connectivity between left mPFC seed and bilateral anterior cingulate cortex (ACC), and between right mPFC seed and bilateral amygdala as compared with HCs (*p* < 0.05, FDRcorr-cluster) (Figures 1A,B and Table 2). OCD patients had reduced FC between right mPFC seed with both left medial superior frontal gyrus (SFG) and bilateral thalamus (*p* < 0.05, FDRcorr-cluster) in comparison with HCs (Figure 1C and Table 2). No other significant group differences in whole-brain FCs were detected for both groups (*ps* > 0.05). The altered FCs were not significantly correlated with any demographical, clinical and head motion variables in each group (*ps* > 0.05, Supplementary Table S2).

**Table 2:** Brain regions showing functional connectivity differences based on default mode network seeds among SCZ and OCD in comparison with HC.

Brain regions	Direction	Voxel	Peak coordinates (*x*/*y*/*z*; MNI)			Peak *T* values	Cohen’s *d*
**SCZ vs. HC**							
PCC as seed	None						
Left mPFC as seed							
Bilateral anterior cingulum cortex (BA32, 24)	SCZ < HC	205	12	24	33	3.75	1.12
Right mPFC as seed							
Bilateral amygdala extending to striatum (BA48, 34)	SCZ < HC	351	−3	−12	−3	4.55	1.36
**OCD vs. HC**							
PCC as seed	None						
Left mPFC as seed	None						
Right mPFC as seed							
Left superior frontal gyrus (BA9)	OCD < HC	163	−12	54	45	4.90	1.47
Bilateral thalamus	OCD < HC	163	9	−15	6	4.24	1.27

*SCZ, schizophrenia; OCD, obsessive-compulsive disorder; HC: healthy control; mPFC, medical prefrontal cortex; PCC, posterior cingulate cortex; BA, Broadmann area; x, y, z, coordinates of peak locations in the Montreal Neurological Institute space (MNI). Significance threshold was set at p < 0.05, FDR cluster level corrected, starting from voxel level p < 0.005 uncorrected.*

### Correlations Between Altered FCs and Sustained Attention Deficits by Group

The correlations between the measurements of sustained attention deficit, that’s IIV, and the altered DMN related FCs were examined in SCZ and OCD group, respectively. None of the demographic and clinical variables were controlled as covariates as none of them were significantly correlated with the IIV or the altered FCs in each patient group. Results showed that in SCZ, the altered FC between left mPFC and bilateral ACC was significantly correlated with their increased IIV (*r* = −0.52, *p* = 0.034) (Figure 1D), and in OCD, the altered FC of right mPFC-left SFG was significantly correlated with their increased variation (*r* = −0.42, *p* = 0.012) (Figure 1E). Correlations between these two FCs and IIV were not significant in the HCs (left mPFC-bilateral ACC: *r* = 0.242, *p* = 0.155; right mPFC-left SFG: *r* = −0.02, *p* = 0.943).

### Common FC Correlated With Sustained Attention in the Whole Patient Sample

**FIGURE 2:**
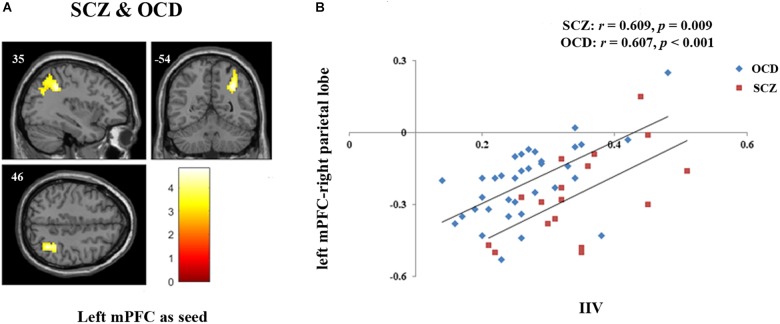
FC associated with sustained attention in both SCZ and OCD. **(A)** Regression model for left mPFC seed including both SCZ and OCD with disease type as covariate revealed that the FC between left mPFC and right parietal lobe [Peak coordinates (*x/y/z*, MNI): 36, –42, 36; 241 voxels; BA 40, peak *T* = 4.83, *p* = 0.005, FDR cluster level corrected, starting from voxel level *p* < 0.005 uncorrected] was significantly correlated with sustained attention indexed by IIV in the whole patient group. **(B)** Further correlation analyses revealed that the extracted values of left mPFC-right parietal lobe FC were significantly correlated with sustained attention in each group (OCD: *r* = 0.607, *p* < 0.001; SCZ: *r* = 0.609, *p* = 0.009).

Regression analyses revealed that the FC between left mPFC seed and right inferior parietal lobe extending to right angular gyrus was significantly correlated with sustained attention in the whole patient sample (*p* = 0.005, FDRcorr-cluster) (Figure 2A). We further extracted the FC values of this cluster and examined the correlation in OCD and SCZ separately. Results showed that the correlation in both groups were significant (OCD: *r* = 0.607, *p* < 0.001; SCZ: *r* = 0.609, *p* = 0.009) (Figure 2B). Values of this cluster were not significantly correlated with IIV in the HCs (*r* = 0.17, *p* = 0.325).

In addition, we evaluated all our effect sizes by comparing which with the required effect size according to the sample sizes to further confirm whether our results were acceptable (Supplementary Text S.1). Also, as half of our patients were medicated, the effects of medication status on our established results of altered SART performance and FCs in SCZ and OCD were further evaluated (Supplementary Text S.2 and Supplementary Table S3).

## Discussion

The current study was aimed to examine whether the DMN connectivity during resting-state can provide information for the performance on sustained attention tasks in both SCZ and OCD. Main results were: (1) both SCZ and OCD patients had enhanced IIV during SART which indicated their poorer sustained attention; (2) reduced resting-state FC of right mPFC-left SFG was significantly correlated with sustained attention deficits in OCD; (3) reduced FC between left mPFC and bilateral ACC was significantly associated with sustained attention dysfunction in SCZ; and (4) the FC between left mPFC and right parietal lobe was significantly correlated with sustained attention in both OCD and SCZ. These results bolster the previous findings of sustained attention problems in both SCZ and OCD patients, and more importantly, they support the hypothesis that the resting-state DMN-involved connectivity can complement information for the changes observed during sustained attention tasks across psychiatric disorders.

In the present study, OCD and SCZ patients had the similar patterns of the impaired behavioral SART performance. Specifically, they both had more omission and commission errors, did not differ in mean RT while demonstrated larger IIV. Compared to more typical attention tasks which have a low go target rate, the SART can capture lapses of attention in a more sensitive way thanks to the design of high target frequency (not 3, 200/225) which allowed monitoring the attention level nearly continuously during the whole task course. Thus, if people have attention fluctuations, it would be reflected on the measurements of IIV, and if the fluctuations were more severe, omission errors occurred. Hence, both omissions errors and the IIV were likely assessments of sustained attention, while the IIV was no doubt the more sensitive one (Molenberghs et al., 2009). Commission errors might be more complex assessments as it might arise from accounts of attention lapses, impaired inhibitory control as well as the speed-accuracy trade-off (Seli, 2016). As in the present study, there were no significance differences in the mean RT, thus the commission errors might mainly reflect impaired inhibitory control and sustained attention deficits in OCD and SCZ patients. Taken together, both our results of enhanced IIV and omission errors in OCD and SCZ indicated their sustained attention deficits, and as the IIV was the more sensitive assessment of sustained attention, we used it in the further correlation analysis, just like some previous studies did (Weissman et al., 2006; Bonnelle et al., 2011; Posner et al., 2016). Notably, according to our power analysis (Supplementary Text S.1), the effect size of IIV in the OCD group (0.632, medium; Cohen, 1988) did not achieve the detectable level (0.674). However, our effect size was comparable to the findings from a meta-analysis, which summarized the cognitive deficits in OCD patients and found that the effect size for sustained attention was 0.499 (medium; Cohen, 1988). Therefore, we might still suggest a moderate difference between OCD patients and HCs in sustained attention cognitive subdomain.

Sustained attention deficits in OCD and SCZ were not novel findings as they were also revealed in many previous studies (Hong et al., 2011; Abramovitch et al., 2013). The most outstanding results in the present study were that the altered resting-state right mPFC-left SFG FC was significantly related with enhanced IIV during SART in OCD and the altered left mPFC-bilateral ACC FC was significantly correlated with sustained attention deficits in SCZ. In addition, the left PFC-right parietal lobe FC was significantly associated with sustained attention in both groups.

SFG is a complex brain region. Previous studies have revealed that it might be parcellated, primarily, into anteromedial, dorsolateral, and posterior subregions with specifically the anteromedial one involvement in the DMN (Li et al., 2013). In order to figure out whether our result of anteromedial SFG is part of DMN, we additionally calculated the FC spatial patterns starting from our SFG cluster and results did show that the maps were largely in line with the previous established templates of DMN (Supplementary Text S.3 and Supplementary Figure S1; Power et al., 2011). This finding might suggest that our altered FC of right mPFC-left SFG in OCD reflected their reduced coupling within DMN. Both clusters of ACC and parietal lobe were submitted to the similar analyses and results showed that the FC maps from ACC was largely consistent with the generally recognized templates of SN (Supplementary Text S.3 and Supplementary Figure S1), and the maps from parietal lobe was largely overlapped with the templates of one of the task-positive networks, i.e., the frontal-parietal network (FPN) (Supplementary Text S.3 and Supplementary Figure S1; Power et al., 2011). Hence, these evidences might indicate our reduced FC of left mPFC-bilateral ACC reflecting impaired interaction between DMN and SN, and FC between left mPFC and right parietal lobe representing interaction between DMN and FPN.

Intact DMN function being important for sustaining a stable attention level could be explained by the following reasons. First, DMN was task-negative, and thus the ability to successful suppression of DMN during tasks was a guarantee of rational reallocation of cognitive resources to the attention demanding processes (Bonnelle et al., 2011; Li et al., 2016). Second, impaired activation of DMN regions usually linked to the mind wandering which was a direct reflect of distraction from focused attention (Hasenkamp et al., 2012). Thus, taken together, these suggested that the impaired coupling within DMN in OCD patients might involved in their sustained attention deficits via reflecting their possible less efficient DMN deactivation or more frequent mind-wandering during SART. Dysfunction of DMN in OCD might also be in line with the clinical observation, that’s OCD patients cannot efficiently recruit this system due to their intrusive and recurrent obsessions. Notably, our finding was not consistent with Posner et al. (2016) which found that the sustained attention deficit in OCD correlated with the FC of mPFC and anterior insula (AI) instead. However, Posner et al. (2016) only used the SD of RT to assess the sustained attention without further adjusting for the differences in RT and they have failed to detect the sustained attention deficits in OCD and HC. Using only the RT SD to assess the sustained attention might make their assessment impure of reflection of RT variation and thus further limit the generalization of their results.

Results for SCZ might be more complex. On the one hand, there were emerging evidences showing that the SN played a key role in controlling switching between DMN and CEN to help DMN and CEN initially adjust an activity level both according to rest and task demands as appropriate. Interestingly, the two hubs in SN had different function focuses. Unlike AI which receives robust sensory input while has very little direct motor input or output connections, the dACC receives little sensory input, while sends strong motor output (Craig, 2009; Menon and Uddin, 2010). Hence, AI may play a more prominent role in receiving and integrating information and while the dACC may be more closely tied to modulating and controlling responses in sensory, motor, and association cortices (Menon and Uddin, 2010). To conclude, these might raise the possibility that the reduced connectivity between ACC and mPFC in SCZ also indicated the inefficient control of SN over DMN which were finally reflected on the improper DMN activity level in SCZ. Hence, these meant that when turning into the cognitive process from the resting-state, the improper DMN activity level might hinder the enough cognitive resources allocated into the sustained attention, which leading to the poor function performance in SCZ. However, as the FC analysis cannot reveal the cause and effect, this hypothesis needs further confirmation. On the other hand, importantly, studies have revealed though DMN was related to the emergence of mind-wandering, the activity in SN also related to the awareness of mind wandering (Hasenkamp et al., 2012). Thus, the impaired interactions between DMN and SN in SCZ might also be involved in the sustained attention via processing of mind-wandering. Attention and concentration scores in SCZ correlated with connectivity in MPFG and dorsal ACC was also revealed in the study of Camchong et al. (2011), which combined with our findings, further supported these regions involved in the sustained attention in SCZ.

Despite these two illness-specific FCs, we also revealed that the FC between left mPFC and a right parietal cluster indicating the interaction between DMN and FPN was correlated with sustained attention in both SCZ and OCD. FPN is one of the prominent task-positive networks which consists of the superior and middle frontal gyrus and the parietal cortex (Power et al., 2011). Previous studies have revealed the involvement of FPN in the top-down attentional and cognitive control, which were vital abilities required in the sustained attention (Sylvester et al., 2012). DMN and FPN typically has the strong anti-relationship (negative correlation). This anti-relationship reflects the cooperation between brain networks to reallocate the attentional resource away from self-referential processing and toward the demands required by the cognitive tasks (Kelly et al., 2008; Pu et al., 2016). Hence, the less negative correlation between DMN and FPN might mean more DMN intrudes upon FPN during the tasks, which limited the attentional sources used in the cognitive control, manifesting in mind-wandering and attention lapse. The correlation between DMN and task-positive networks competitive relationship and sustained attention was also revealed by Kelly et al. (2008). Notably, in their study, they found that the relationship existed both at resting state and during task performance, which further support the utility of resting-state FC in complementing information for cognitive function. Moreover, the correlation between left-mPFC-right parietal lobe FC and sustained attention was existed in both SCZ and OCD. This raises an interesting question on whether or not this relationship is transdiagnostic across other different psychiatric disorders. We would like to test this hypothesis in the future.

In this study, we found both the FC within DMN and between DMN and task-positive networks serving as neural correlates of sustained attention. This might be in line with the acknowledgment that the networks did not function independently in the brain (Menon, 2011). The dysfunction of a network may be reflected in both coupling within itself and interactions between it and other networks, and both the coordination of FC within and between networks is important for behavior. In addition, although we found the transdiagnostic neural correlate of sustained attention in both SCZ and OCD, i.e., the FC between left mPFC and right parietal lobe, the illness-specific neural underpinnings were also revealed. This might be related to the different resting-state FC baselines in different disorders. For example, the OCD was usually found to have reduced DMN connectivity while the SCZ patients usually demonstrated DMN hyper-connectivity (Jang et al., 2010; Hu et al., 2017). Thus, different altered resting-state baselines might account for mechanism of different specific FCs involved in the sustained attention deficits.

Task-induced deactivation of DMN or altered inter-DMN FC were consistently found to play an important role across a wide variety of cognitive tasks, which raised the doubts on whether our detected altered neural correlates of FCs were independent of the specific cognitive processes (Sheffield and Barch, 2016). In terms of this, though we could not totally rule out this possibility, we thought it’s important to note that Mayer et al. (2010) compared the common and selective deactivation patterns in response to working memory tasks and attention tasks, and results interestingly showed that the DMN can be subdivided into common deactivate regions and selective deactivate areas which depended on the specific tasks. More importantly, they found that areas suppressed only in attention task were located in the superior and inferior frontal gyrus, medial frontal gyrus, ACC and paracentral lobule. Our results of sustained attention related regions were quite overlapped with the aforementioned regions, which raised the possibility that the altered connectivity significantly correlated with sustained attention detected in the current study might depend on specific tasks. However, we did not include other cognitive tasks in the present study. Thus, whether the FC revealed in the current study underlying mechanisms shared across cognitive functions or just contributed to the sustained attention needs further clarification.

This study has several limitations. First, our results were based on the resting-state data. Further task-based studies are required to confirm the role of altered within and between DMN connectivity in the sustained attention in OCD and SCZ. Second, though the correlation between altered FC and sustained attention deficits were all reached the medium level, the significance in the SCZ cannot survive the strict Bonferonia correction (0.05/2). These might be related to our relatively small sample size of SCZ sample and future analysis conducted in larger samples are warranted. Third, movement is a potential confound, the effects of which cannot be completely removed. However, it’s unlikely to account for our findings, given that the patient groups and HCs were not differed in terms of head motion, and the identified FCs were not significantly correlated with FD in the present study (Supplementary Table S2). Forth, some of our patients were taking medications. Similarly, the effects of medication cannot be clearly ruled out, although it is also unlikely to account for our results (Supplementary Text S.2 and Supplementary Table S3). Future studies with better design or conducted in the drug-naive samples are needed to clarify the effects of medication on sustained attention as well as the FCs. Last but not least, significance threshold of *p* < 0.005 voxel level uncorrected combined with *p* < 0.05 cluster level FDR corrected was adopted to correct multiple comparisons in the present study. Though this correction level was acceptable based on the previous studies (Ko et al., 2015; Wagner et al., 2017), it was not that strict to some extent. Thus, we might suggest that it should be cautious to generalize our results and further replication studies are needed.

## Conclusion

In summary, the present study provides evidence for the utility of resting-state FC to account for sustained attention deficits seen in the psychiatric disorders. Transdiagnostically, the FC between left mPFC and right parietal lobe which indicated the interaction between DMN and FPN was associated with sustained attention in both SCZ and OCD. Disorder-specifically, for patients with OCD, the reduced resting-state FC of right mPFC-left SFG which indicated impaired coupling within DMN was neural correlates of their impaired sustained attention. For patients with SCZ, the reduced resting-state FC of left mPFC-bilateral ACC indicating altered interaction between SN and DMN seemed to be involved in their enhanced attention fluctuations. Findings suggest that the coordination of FC both within DMN and between DMN and task-positive networks is important for sustained attention. The reason that the illness-specific correlates are revealed may be due to the different resting-state dysfunction baselines in different psychiatric disorders.

## Author Contributions

XZ designed the study. JF analyzed the data and wrote the paper. JG, WL, HL, and HZ collected the data. MZ, JY, RC, and CT advised on the interpretation of the results and contributed to the final draft.

## Conflict of Interest Statement

The authors declare that the research was conducted in the absence of any commercial or financial relationships that could be construed as a potential conflict of interest.
