# Supplementary material for: Resting-State Default Mode Network Related Functional Connectivity Is Associated With Sustained Attention Deficits in Schizophrenia and Obsessive-Compulsive Disorder
Source: Front Behav Neurosci. 2018 Dec 19;12:319. doi: 10.3389/fnbeh.2018.00319 (PMC6305719; doi:10.3389/fnbeh.2018.00319)
Supplement: Supplementary file 1 [file Table_1.docx]

**Table S1** Tests of normal distribution for continuous variables in three groups separately.

|  | **Age** | **Educa**  **-tion** | **BDI** | **STAI**  **_S** | **STAI**  **_T** | **Omission errors** | **Commi**  **-ssion errors** | **meanRT** | **Dura-tion** | **IIV** | **Illness severity** | | | | | |
| --- | --- | --- | --- | --- | --- | --- | --- | --- | --- | --- | --- | --- | --- | --- | --- | --- |
| ***SCZ*** |  |  |  |  |  |  |  |  | |  | **PANSS**  **_P** | **PANSS**  **_N** | | **PANSS**  **_G** | | **PANSS**  **_T** |
| Kolmogorov  -Smornov Z | 0.76 | 1.18 | 0.47 | 0.59 | 0.48 | 0.94 | 0.72 | 0.41 | 0.93 | 0.53 | 0.69 | 0.74 | | 0.44 | | 0.50 |
| *p* | .612 | .124 | .980 | .873 | .975 | .343 | .672 | .996 | .355 | .944 | .722 | .640 | | .989 | | .962 |
|  |  |  |  |  |  |  |  |  | |  | | |  | |  | |
| ***OCD*** |  |  |  |  |  |  |  |  | | **YBOCS_O** | | | **YBOCS_C** | | **YBOCS_T** | |
| Kolmogorov  -Smornov Z | 0.87 | 1.09 | 0.64 | 0.46 | 0.90 | 1.74 | 1.04 | 0.92 | 1.15 | 1.08 | 0.93 | | 0.58 | | 0.53 | |
| *p* | .437 | .188 | .811 | .986 | .389 | < .01 | .234 | .365 | .141 | .193 | .356 | | .892 | | .945 | |
|  |  |  |  |  |  |  |  |  | |  |  | |  | |  | |
| ***HC*** |  |  |  |  |  |  |  |  | |  |  | |  | |  | |
| Kolmogorov  -Smornov Z | 1.04 | 1.03 | 1.19 | 0.53 | 0.77 | 1.96 | 0.75 | 1.33 | - | 1.11 | - | | - | | - | |
| *p* | .229 | .237 | .119 | .939 | .602 | < .005 | .622 | .058 | - | .171 | - | | - | | - | |

SCZ, schizophrenia; OCD, obsessive-compulsive disorder; HC: healthy control; SART, sustained attention to response task; SD: standard deviation; YBOCS, Yale-Brown Obsessive-Compulsive Scale; PANSS, Positive and Negative Syndrome Scale; BDI, Beck Depression Inventory; STAI, State Trait Anxiety Inventory; RT, reaction time; IIV, intra-individual variation.

**Table S2** Correlations between demographic, clinical and head motion variables and deficits during SART as well as altered FCs in patient groups [r (*p*)].

| **Variables** | **Age** | **Educa**  **-tion** | **Illness severity** | | | | | | | **Duration** | **BDI** | **STAI_S** | **STAI_T** | **FD** |
| --- | --- | --- | --- | --- | --- | --- | --- | --- | --- | --- | --- | --- | --- | --- |
| ***SCZ*** |  |  | **PANSS_P** | **PANSS_N** | | **PANSS_G** | | **PANSS_T** | |  |  |  |  |  |
| Omission error | 0.11(.674) | 0.15(.570) | -0.17(.523) | 0.32(.233) | | 0.29(.280) | | 0.20(.456) | | -0.36(.171) | 0.14(.619) | 0.05(.847) | 0.01(.960) | - |
| Commission error | 0.29(.268) | 0.21(.423) | -0.06(.811) | 0.04(.884) | | -0.03(.911) | | -0.03(.916) | | -0.62^**^(.009) | 0.09(.973) | 0.22(.391) | 0.29(.253) | - |
| IIV | -0.12(.649) | 0.01(.993) | -0.12(.639) | 0.40(.111) | | 0.04(.886) | | 0.10(.709) | | -0.32(.215) | 0.01(.973) | 0.38(.134) | 0.31(.232) | - |
| Left mPFC-bilateral ACC | 0.40(.112) | 0.26(.312) | -0.23(.369) | -0.44(.076) | | -0.14(.588) | | -0.29(.254) | | 0.08(.774) | 0.12(.635) | -0.12(.641) | 0.10(.704) | 0.18(.487) |
| Right mPFC-bilateral amygdala | -0.42(.098) | -0.21(.428) | 0.09(.732) | 0.08(.756) | | 0.19(.471) | | 0.17(.525) | | 0.49(.057) | -0.01(.984) | 0.17(.528) | 0.04(.882) | 0.09(.739) |
| Left mPFC-right parietal lobe | 0.16(.539) | 0.09(0.734) | -0.14(.595) | 0.99(.706) | | -0.10(.712) | | -0.08(.772) | | -0.34(.186) | 0.15(.571) | 0.32(.216) | 0.44(.078) | -0.14(.582) |
| ***OCD*** |  |  | **YBOCS_O** | | **YBOCS-C** | | **YBOCS-T** | |  | |  |  |  |  |
| Omission error^*^ | -0.13(.463) | -0.20(.256) | 0.05(.780) | | 0.20(.262) | | 0.20(.253) | | -0.08(.670) | | -0.01(.974) | -0.08(.665) | 0.01(.979) | - |
| Commission error | 0.01(.940) | 0.23(.179) | -0.05(.792) | | -0.01(.938) | | -0.03(.857) | | -0.02(.925) | | 0.09(.598) | -0.11(.543) | -0.03(.858) | - |
| IIV | -0.06(.745) | 0.20(.239) | -0.03(.875) | | 0.01(.981) | | -0.01(.960) | | -0.18(.302) | | 0.24(.161) | 0.19(.272) | 0.12(.506) | - |
| Right mPFC-left SFG | -0.05(.768) | -0.03(.850) | -0.03(.856) | | -0.14(.415) | | -0.13(.457) | | -0.06(.728) | | -0.04(.850) | 0.09(.600) | -0.07(.688) | 0.01(.956) |
| Right mPFC-bilateral thalamus | -0.03(.884) | 0.07(.688) | 0.16(.371) | | -0.08(.641) | | 0.01(.985) | | -0.06(.745) | | -0.18(309) | -0.08(.633) | -0.22(.211) | -0.15(.387) |
| Left mPFC-right parietal lobe | -0.20(.269) | -0.01(.963) | 0.11(.548) | | 0.02(.926) | | 0.06(.729) | | -0.28(.127) | | 0.29(.105) | 0.20(.272) | 0.14(.432) | 0.15(.412) |

* The correlations between omission error and other variables in OCD were indicated by spearman correlation as the distribution of omission error in OCD didn’t comply with the normal distribution. All other correlations were calculated by Pearson correlation.

SCZ, schizophrenia; OCD, obsessive-compulsive disorder; HC: healthy control; SART, sustained attention to response task; SD: standard deviation; FD, framewise displacement; YBOCS, Yale-Brown Obsessive-Compulsive Scale; PANSS, Positive and Negative Syndrome Scale; BDI, Beck Depression Inventory; STAI, State Trait Anxiety Inventory; RT, reaction time; IIV, intra-individual variation; mPFC, medical prefrontal cortex; PCC, posterior cingulate cortex; FC, functional connectivity; ACC, anterior cingulum cortex; SFG, superior frontal gyrus.

**S.1** To further confirm whether our results were acceptable, we calculated the required effect size according to our sample sizes. Results showed that for our comparisons of SCZ *vs.* HC and OCD *vs.* HC, to achieve 80% power at 5% significance level, the required effect sizes (Cohen’s *d*) would be 0.84 and 0.67 respectively (G-power, version 3.1.9.2). As shown in **Table 1** and **Table 2**, The Cohen’s *d* for the SCZ group in the present study ranged from 0.93 to 1.47, which were all larger than the detectable effect size (0.840), suggesting that the results were acceptable. In terms of the OCD group, all the effect sizes for the altered FCs and the impaired omission errors were larger enough to be sound (ranged from 0.69 to 1.47), however, the Cohen’s *d* of IIV (0.632) and commission errors (0.485) were not reached that required level.

**S.2** In order to figure out whether medication status had effect on performance on the SART deficits and the altered FCs revealed in the SCZ and OCD, we further compared the demographical and clinical variables, SART performance and the altered FCs values between the medicated and unmediated groups in SCZ and OCD separately. Results showed that in terms of SCZ, patients who was not under treatment had trend-level higher scores in the symptom severity indexed by the PANSS total scores (*p* = 0.056), which was mainly driven by the higher scores in the General Psychopathology subscale (*p* = 0.006). No other group differences in demographical and clinical variables were detected (*ps* ≥ 0.05). In both samples, medicated and unmedicated groups did not differ in any of the measurements in SART and the altered FCs (*ps* ≥ 0.05, see details in **Table S3**). Thus, in both SCZ and OCD, medicated and unmedicated patients had comparable alterations in both SART performance and FCs, indicating that the dysfunctions in each psychiatric condition were not likely driven by one single subgroup. For the SCZ sample, though patients with medications were trend-level severer in symptom presentation, the FCs and sustained attention did not differ between two subgroups, and were not significantly correlated with illness severity (**Table S2**), which suggested that at least, our findings were not likely to be affected by the slightly differences in severity between two subgroups.

**Table S3** The effect of medication status on measurements of sustained attention in SCZ and OCD.

| **Domain** | ***SCZ*** | | | |  |  | ***OCD*** | | | | |
| --- | --- | --- | --- | --- | --- | --- | --- | --- | --- | --- | --- |
|  | **Medicated**  **(*N = 11*)** | **Unmedicated**  **(*N = 6*)** | **t/χ2** | ***p*** | | **Medicated**  **(*N = 15*)** | | **Unmedicated**  **(*N = 20*)** | ***t**** | ***p*** |  |
| age | 22.09(3.88) | 23.00(3.74) | -0.47 | 0.647 | | 23.40(5.73) | | 24.20(5.42) | -0.42 | 0.676 |  |
| Sex (female, %) | 4(33.4) | 4(66.7) | 1.43 | 0.232 | | 8(53.3) | | 8(40.0) | 0.61 | 0.433 |  |
| Education (years) | 13.45(2.52) | 14.33(3.14) | -0.67 | 0.513 | | 15.00(2.78) | | 14.93(1.99) | 0.09 | 0.926 |  |
| Duration (month) | 18.36(15.67) | 22.83(33.77) | -0.38 | 0.711 | | 61.67(71.68) | | 61.25(58.41) | 0.02 | 0.985 |  |
| PANSS_Total | 54.54(11.10) | 66.33(10.67) | -2.12 | 0.056 | | - | | - | - | - |  |
| PANSS_P | 17.63(4.06) | 17.17(4.67) | 0.22 | 0.831 | | - | | - | - | - |  |
| PANSS_N | 9.36(2.87) | 12.00(4.29) | -1.53 | 0.149 | | - | | - | - | - |  |
| PANSS_G | 27.54(5.61) | 31.17(5.42) | -3.42 | 0.006 | | - | | - | - | - |  |
| YBOCS_Total | - | - | - | - | | 32.13(6.16) | | 29.05（6.13） | 1.50 | 0.144 |  |
| YBOCS_O | - | - | - | - | | 16.80（2.57） | | 16.10（2.94） | 0.74 | 0.467 |  |
| YBOCS_C | - | - | - | - | | 15.33（5.25） | | 12.95（4.55） | 1.44 | 0.160 |  |
| BDI | 19.54(8.99) | 15.50(11.22) | 0.81 | 0.428 | | 18.67（10.40） | | 21.10（9.51） | -0.72 | 0.477 |  |
| STAI-T | 52.91(12.21) | 53.17(7.47) | -0.05 | 0.963 | | 57.27（9.62） | | 58.40（8.20） | -0.38 | 0.710 |  |
| STAI-S | 51.64(14.74) | 47.33(12.40) | 0.61 | 0.554 | | 48.87（11.99） | | 54.65（10.78） | -1.50 | 0.144 |  |
| Omission errors | 0.04(0.05) | 0.04(0.03) | 0.16 | 0.875 | | 0.03(0.05) | | 0.02(0.03) | 0.70 | 0.488 |  |
| Commission errors | 0.49(0.24) | 0.45(0.22) | 0.32 | 0.755 | | 0.35(0.21) | | 0.44(0.27) | -0.97 | 0.339 |  |
| Mean RT | 414.82(67.09) | 377.41(94.38) | 0.95 | 0.355 | | 425.87(95.97) | | 369.18(106.58) | 1.62 | 0.114 |  |
| IIV | 0.35(0.08) | 0.33(0.10) | 0.39 | 0.699 | | 0.26(0.07) | | 0.32(0.14) | -1.45 | 0.158 |  |
| Left mPFC-bilateral ACC | -0.27(0.14) | -0.21(0.15) | -0.94 | 0.362 | | - | | - | - | - |  |
| Right mPFC-bilateral amygdala | -0.06(0.09) | -0.002(0.10) | -1.23 | 0.238 | | - | | - | - | - |  |
| Right mPFC-left SFG | - | - | - | - | | 0.31(0.19) | | 0.45(0.21) | -2.06 | 0.072 |  |
| Right mPFC-bilateral thalamus | - | - | - | - | | 0.03(0.17) | | -0.06(0.14) | 1.67 | 0.104 |  |
| Left mPFC-right parietal lobe | -0.27(0.21) | -0.24(0.15) | -0.27 | 0.794 | | -0.23 (0.11) | | -0.25(0.18) | 0.49 | 0.626 |  |

SCZ, schizophrenia; OCD, obsessive-compulsive disorder; RT, reaction time; IIV, intra-individual variation; mPFC, medical prefrontal cortex; PCC, posterior cingulate cortex; ACC, anterior cingulum cortex; SFG, superior frontal gyrus.

**S.3** In order to verify whether we can interpret our results of SFG cluster as part of the DMN, ACC cluster as part of SN and parietal lobe as part of FPN, we calculated the mean FCs starting from these ROIs. One-sample *t* tests with significance threshold set at *p* < 0.01, FDR corrected were used to examine the spatial maps for ROI by group.

The respective spatial pattern was shown in **Figure. S1**. Results revealed that both spatial patterns of SFG in OCD and HCs consisted of mainly the mPFC, PCC and the angular, which almost matched the classical templates of DMN (Allen et al., 2011; Manoliu et al., 2014). Spatial patterns of ACC in SCZ and HCs included primarily the inferior frontal gyrus, AI and dorsal ACC, which were in line with the templates of SN established previously (Allen et al., 2011; Manoliu et al., 2014). Spatial patterns of parietal lobe in SCZ and OCD included mainly the middle and superior frontal gyrus and parietal lobes, which were largely overlapped with the classical templates of FPN (Power et al., 2011). Hence, we might propose that the interpretation of SFG as part of DMN, ACC as part of the SN and parietal cluster as part of FPN was proper and reasoned.


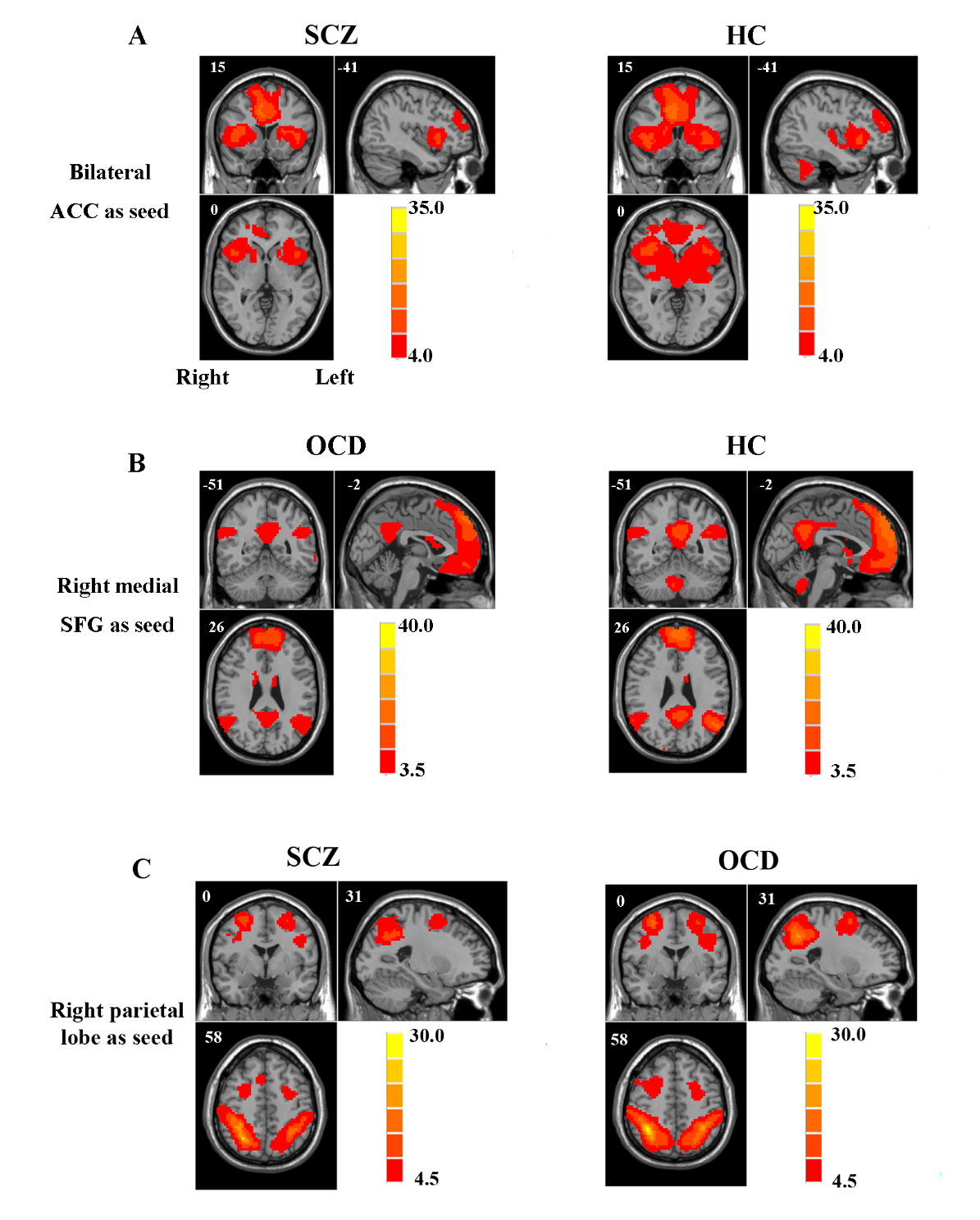


**Figure. S1 (A)** Spatial patterns starting from cluster of bilateral ACC for SCZ and HC group respectively. **(B)** Spatial patterns starting from cluster of left SFG for OCD and HC group respectively. (**C**) Spatial patterns starting from cluster of right parietal cluster for SCZ and OCD respectively. One-sample *t*-test, *p* < 0.01, false discovery rate (FDR) corrected.

***Reference***

Allen, E. A., Erhardt, E. B., Damaraju, E., Gruner, W., Segall, J. M., & Silva, R. F., et al., 2011. A baseline for the multivariate comparison of resting-state networks. Frontiers in Systems Neuroscience, 5(5), 2.

Manoliu, A., Riedl, V., Zherdin, A., Mühlau, M., Schwerthöffer, D., & Scherr, M., et al., 2014. Aberrant dependence of default mode/central executive network interactions on anterior insular salience network activity in schizophrenia. Schizophr Bull., 40(2), 428-437.

Power, J. D., Cohen, A. L., Nelson, S. M., Wig, G. S., Barnes, K. A., Church, J. A., ... & Petersen, S. E. (2011). Functional network organization of the human brain. Neuron, 72(4), 665-678.
